# Characterizing the Effect of Freeze–Thaw Cycling on Pore Structure of Asphalt Concrete Mixtures Using X-ray CT Scanning

**DOI:** 10.3390/ma16186254

**Published:** 2023-09-18

**Authors:** Mai Alawneh, Haithem Soliman, Ania Anthony

**Affiliations:** 1 Department of Civil, Geological, and Environmental Engineering, University of Saskatchewan, 57 Campus Drive, Saskatoon, SK S7N 5A9, Canada; maa592@usask.ca; 2 Saskatchewan Ministry of Highways, 126-105th Street East, Saskatoon, SK S7N 1Z3, Canada; ania.anthony@gov.sk.ca

**Keywords:** asphalt concrete, freeze–thaw (F–T) cycles, X-ray computer tomography (CT) scan fine aggregate matrix (FAM), air voids, polymer-modified binder

## Abstract

Freeze–thaw (F–T) cycling presents a challenge when building durable pavement structures in cold regions. Understanding the changes within the microstructure of asphalt concrete (AC) due to F–T conditions is crucial for developing a resilient pavement design. This study investigates the impact of F–T cycles on five AC mixtures using X-ray computed tomography (CT) scanning. Image analysis was completed to evaluate the changes in the microstructure of the AC samples before and after exposure to 30, 60, and 90 F–T cycles. The changes/degradation in the microstructure were evaluated based on analyzing the distribution and properties of air voids within the AC samples. The results showed that an X-ray CT scan can successfully capture the impact of F–T cycles on the structure of air voids in different AC mixtures. The findings of this research provide guidelines for understanding the mechanism of F–T damage within AC, which can assist in optimizing the performance of AC in cold regions.

## 1. Background

Roadways provide safe and reliable transportation networks for the movement of people and goods. Asphalt concrete (AC) is widely used by transportation agencies in roadway construction due to its cost-effectiveness and ease of maintenance. However, in cold regions, AC is subjected to the detrimental effects of freeze–thaw (F–T) cycles. The repetitive F–T process causes significant stresses within AC, which leads to surface distress and reduction in roadway service life [1,2].

**Figure 1:**
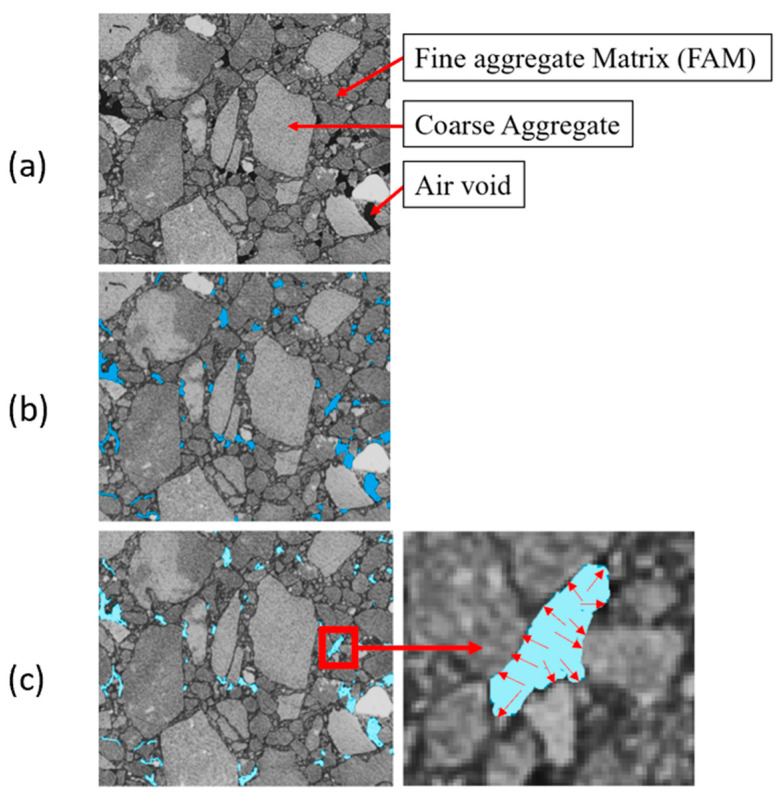
Freeze–thaw damage initiation in AC: (**a**) AC components; (**b**) Infiltrated water within AC voids; (**c**) Formation of ice crystals within AC voids.

AC is a composite material that consists of coarse aggregates, fine aggregate matrix (FAM), and air voids (see Figure 1a). The FAM represents fine aggregates, asphalt binders, and fillers. When exposed to moisture conditions, water infiltrates through the permeable voids in the AC mix (the blue areas in Figure 1b). At freezing temperatures, the infiltrated water forms ice crystals (light blue areas in Figure 1c) with a larger volume, which causes an internal pressure/stress within the AC mix (red arrows in Figure 1c). As the temperature rises above freezing, the ice crystals melt and turn to water again. Exposure of AC to repetitive F–T cycles can cause distress and loss of structural integrity.

An asphalt binder is a viscoelastic material with temperature-dependent properties. An asphalt binder needs to maintain good adhesion with coarse aggregate particles and cohesion within the FAM. At cold temperatures, the asphalt binder becomes stiffer, which can affect its ability to provide effective adhesion and cohesion within the AC mix [3]. Therefore, selection of a suitable asphalt binder grade is important to maintain the integrity and improve the long-term performance of AC pavements in cold regions.

The presence and distribution of air voids within AC mixtures play a crucial role in their F–T performance. Advancements in non-destructive testing techniques have paved the way for novel approaches to studying the evolution of air voids within AC. Recently, X-ray computed tomography (CT) scanning has emerged as a powerful and non-invasive tool to capture intricate details of the internal structure of AC [4].

Several studies have explored the impact of F–T cycling on the mechanical behavior of AC using conventional mechanical tests. Fing et al. [5] studied the F–T durability of AC mixtures with different gradations. Freeze–thaw damage was observed in terms of mechanical strength reduction, volume expansion, and rapid deterioration due to loss of adhesion. Dense-graded mixtures had the best freeze–thaw stability, while semi-open-graded mixtures performed the worst.

Mauduit et al. [6] investigated the impact of F–T cycling on partially water-saturated AC samples. Laboratory tests showed significant swelling at the start of freezing periods for highly saturated samples, which can lead to detrimental effects on pavement in areas with varying moisture contents. Another study by Lamothe et al. [7] examined the impact of F–T cycling on AC under dry and partially saturated conditions. The study evaluated axial strain during F–T cycles, the linear coefficient of thermal expansion (LCTE), dilation from ice formation, and contraction due to ice melting. Results show that changes in these parameters are more significant for partially saturated samples with water or brine.

Tarefder et al. [8] used the Bending Beam Rheometer (BBR) test to assess the influence of F–T cycling on the fatigue life of asphalt binders and pavements. The results showed that the degradation in asphalt binders caused by F–T cycles can reduce the overall stiffness and fatigue life of pavement structures. Ozgan et al. [9] revealed that the Marshall Stability value of the AC mixture was lowered by 63.8% and the air voids were increased by 40% after being exposed to F–T cycles for 24 days. Stasiuk et al. [10] evaluated the performance of various tack coat products using the interlayer shear strength (ISS) test under F–T cycling in cold climates. Results showed that the type of tack coat material and F–T cycling affect the failure mode of the AC samples, which can result in a delamination between AC lifts.

Imaging techniques have been employed by researchers for a better understanding of the F–T damage mechanism in AC. Obaidat et al. [11] utilized a normal-based camera and Silicon Video Image Processing (SVIP) software [M1] to map and digitize the surface of AC mixtures subjected to F–T cycles. Results showed that the used imaging technique can predict the stripping potential of the AC mixtures. 

Xu et al. [12,13] utilized X-ray CT scanning to analyze the microstructure of AC mixtures after F–T conditioning. Three types of AC mixtures were used for the test: a conventional dense-graded asphalt mixture with 80/100 penetration grade asphalt, a stone mastic asphalt mixture with 80/100 penetration grade asphalt, and an open-graded asphalt mixture with a rubberized asphalt binder of 80 grades. The results showed degradation in the asphalt-aggregate bond due to F–T cycling, formation of new air voids, and enlargement of existing voids, which can affect the durability of pavement. The internal structure varied among the three AC types, leading to different patterns of internal structure evolution and the development of air voids and micro-cracks after F–T cycles.

Zhang et al. [14] combined F–T cycling and microwave heating to investigate the healing process of two types of AC mixtures (AC-13 and OGFC-13). The research determined the optimal timing for healing two types of AC mixtures when moisture damage reaches specific levels. X-ray CT technology was used to scan and assess the internal structure, followed by digital image processing for analysis. The results demonstrated that F–T damage and microwave heating changed the internal structure by affecting the pore characteristics. The F–T cycles primarily increased the count of effective pores, while microwave healing primarily raised the count of ineffective pores.

A recent study by Yang et al. [15] explored the use of recycled steel slag in asphalt pavements, particularly its performance in harsh freeze–thaw conditions. It introduced three types of fibers (basalt, polyester, and lignin) and rubber powder into the steel slag asphalt mixture (SSAM) to enhance its durability. Optimal bitumen and fiber content were determined through a robust methodology, and X-ray (CT) was used to assess air voids. The study revealed that incorporating fibers improves SSAM’s resistance to F–T damage and enhances moisture stability.

In this study, an X-ray CT scan was used to capture and analyze the distribution of air voids for five AC mixtures before and after subjecting them to saturation and 30, 60, and 90 F–T cycling. Previous studies showed that the structure of air voids depends on the composition of the AC mixture. In this study, the tested AC mixtures had different contents of reclaimed asphalt pavement (RAP) and different types of binders (unmodified, polymer-modified, and crumb-rubber modified), which have not been covered in the literature. The changes in the size and distribution of air voids due to F–T cycling were characterized based on the formation of new air voids, enlargement of pre-conditioning air voids, and changes in the distribution of air voids. The change in the characteristics of air voids can be used as an indicator for the performance of AC mixtures in cold regions with F–T conditions.

The findings of this research contribute to the understanding of the mechanism of F–T damage in AC and offer guidance for the mix design and material selection. The knowledge and methodology presented in this paper can be adopted by other researchers to characterize the behavior of AC under different loading and environmental conditions.

## 2. Materials and Methods

### 2.1. Samples Preparation

Five loose samples of AC mixtures were collected from actual construction projects by the Ministry of Highways in Saskatchewan, Canada. The samples represent a conventional AC mixture (Mix 1); an AC mixture with polymer-modified binder and 20% of Recycled Asphalt Pavement (RAP) (Mix 2); an AC mixture with 55% of RAP (Mix 3); an AC mixture with crumb-rubber-modified binder, containing 17.5% crumb rubber by weight of binder (Mix 4); and an AC mixture with 8% of RAP (Mix 5). Table 1 provides a summary of the properties of the five AC mixtures.

**Figure 2:**
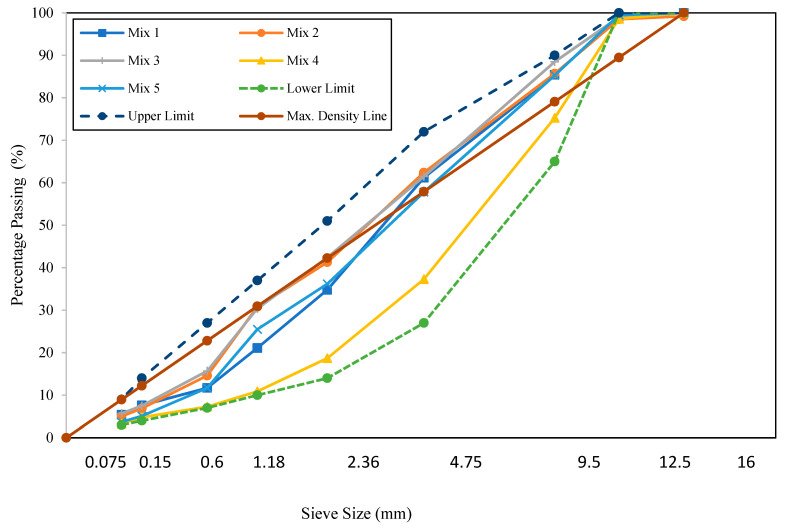
Aggregate gradations for the test AC mixtures (0.45 power chart).

Figure 2 shows the aggregate gradations for the five AC mixtures. All aggregate gradations were within the specification limits. Mix 4 had a coarser aggregate gradation than the other mixtures, with a gradation comparable to the lower limit of the specifications. Mix 1 and Mix 5 had aggregate gradations that represent the average of the specification limits, with Mix 5 having more medium sand than Mix 1. Mix 2 and Mix 3 had finer aggregate gradations than the other mixtures, with more sand materials in the aggregate composition. Mix 3 with 55% of RAP was a trial mix that the Ministry of Highways used in the construction of a test section as part of their efforts to increase the use of recycled materials and preserve natural resources.

The loose AC materials were heated and remixed to achieve the required compaction temperature, as listed in Table 1. A set of compacted samples was prepared for each AC mixture according to ASTM D6925-15 [16]. A Superpave Gyratory Compactor (SGC) was used for compaction with an angle of gyration of 1.25°, a vertical pressure of 0.6 MPa, and a gyration speed of 30 rpm. The compacted cylindrical samples measured 150 mm in diameter and 150 mm in height.

### 2.2. Verification of Volumetric Properties

The volumetric properties of the compacted samples were measured and compared with each respective mix design. The bulk specific gravity (G_mb_) and theoretical maximum specific gravity (G_mm_) were measured according to ASTM D2726/D2726M-19 [17] and ASTM D2041/D2041M-19 [18], respectively. Volumetric analysis was completed to calculate voids in mineral aggregate (VMA), voids filled with asphalt (VFA), and air voids (VA) for the compacted samples. Table 2 summarizes the specific gravities and volumetric properties of the AC mixtures. There are minor differences between mix design values and laboratory values, which can be attributed to typical variabilities in AC materials. In addition, the laboratory samples were prepared using loose mixtures collected for actual construction projects, not laboratory-prepared mixtures, which may have contributed to these differences. However, these differences are acceptable for AC materials.

### 2.3. X-ray CT Scan Samples

**Figure 3:**
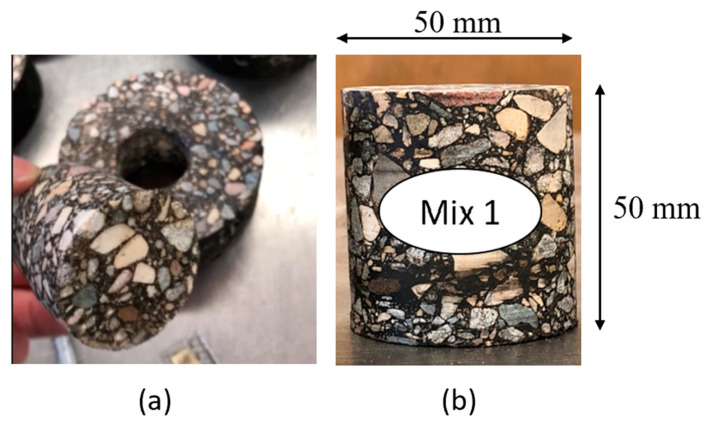
AC sample for X-ray CT scan: (**a**) sample extraction; (**b**) the dimensions of the extracted sample.

Small cylindrical cores measuring 50 mm in diameter and 50 mm in height were extracted from the lab-compacted samples, as shown in Figure 3. The small cores from each AC mixture were scanned using an X-ray CT scanning system. The small cores were scanned before any conditioning took place to evaluate their initial structure. Each small core was scanned again after being exposed to moisture saturation and after each of the three levels of F–T conditioning (30, 60, and 90 F–T cycles).

### 2.4. Samples Conditioning

Before any conditioning, the dimensions and dry weight (A) of each AC sample were measured. The volume of air voids (Va) in cubic centimeters was calculated using Equation (1). Samples were subjected to vacuum saturation by immersing the samples in a sealed chamber filled with distilled water and applying a vacuum pressure of 70 kPa for 30 min. The vacuum pressure was reduced after 30 min from 70 kPa to 10 kPa according to a modified AASHTO T 283 procedure [19]. After two hours, the samples were removed from the chamber. The saturated surface dry (SSD) weight (B’) was measured for each sample according to AASHTO T 166 [20].

The volume of absorbed water in cubic centimeters (J) and degree of saturation (DS) were calculated using Equation (2) and Equation (3), respectively. The saturated samples were wrapped with plastic wrap to keep them sealed and avoid any water loss. The previous steps were repeated for all samples after each X-ray CT scan and F–T conditioning stage.

Va = (Pa·V)/100(1)

where V is the sample volume in cubic centimeters.

Pa is a percentage of air void.

J = B’ − A(2)

where B’ is the SSD weight of the sample in grams.

A is the dry sample’s weight in the air in grams.

DS = (100 J)/Va(3)

where DS is the sample degree of saturation (percent).

**Figure 4:**
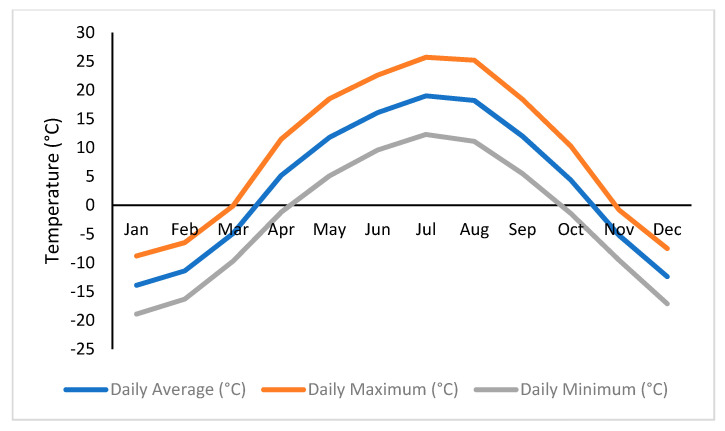
Average minimum and maximum temperatures for Saskatoon from 1981 to 2010 (source: Environment Canada, climate.weather.gc.ca).

**Figure 5:**
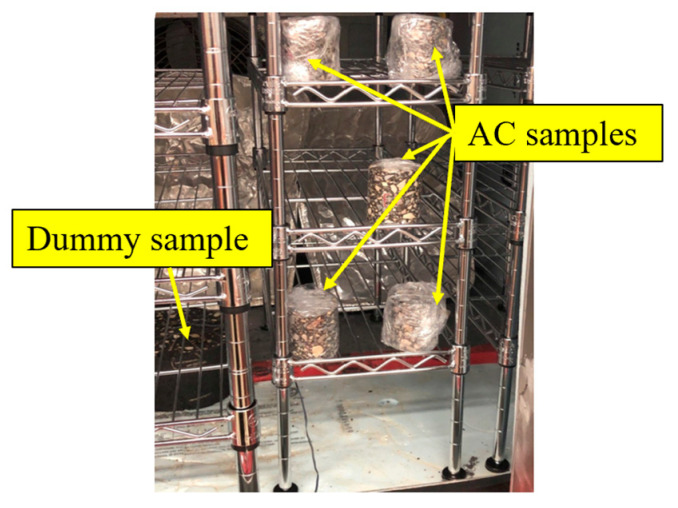
F–T cycling of the AC samples.

The saturated samples were placed in an environmental chamber and exposed to F–T cycling. Each F–T cycle consists of 12 h freezing phase at −20 °C followed by 12 h thawing phase at 20 °C. These temperatures were selected based on historical temperature data for the study region, as shown in Figure 4. The temperatures were also selected to ensure that samples can reach complete freezing and thawing under the accelerated laboratory conditions. A dummy sample was placed in the middle of the chamber with a thermocouple embedded inside the sample to monitor the core temperature of samples during F–T cycling, as shown in Figure 5.

### 2.5. X-ray CT Scanning and Digital Image Processing

**Figure 6:**
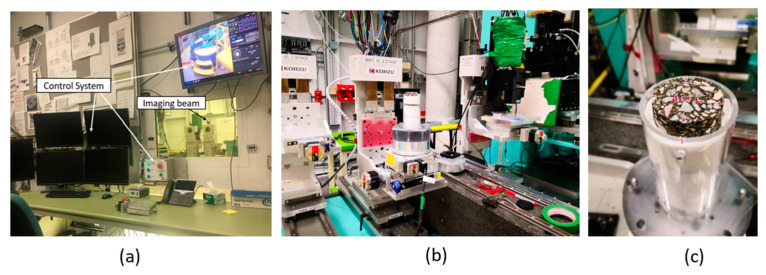
(**a**) BMIT-ID beamline and controls; (**b**) AC sample in the BMIT-ID beamline; (**c**) AC sample inside the mold for the X-ray CT scan.

X-ray CT imaging was used to scan the internal structure of the AC mixtures with high-resolution images showing fine details. The X-ray CT system consists of two main parts: an X-ray source and a detector that measures the intensity of the X-rays after passing through the sample. The recorded intensities were transformed into a gray-scale map, which shows the internal structure of AC based on component densities. Higher-density objects, such as coarse aggregates, have a brighter color, while lower-density objects, such as air voids, have a darker color. Asphalt binder falls in between and shows in grey tones. The collected images were subjected to multiple steps of digital image processing to enhance their quality, remove noise, and extract the data of interest from the images. X-ray CT imaging was conducted at the Biomedical Imaging and Therapy Facility (BMIT)—Insertion Device (ID) beamline at the Canadian Light Source (CLS), Canada, as shown in Figure 6.

**Figure 7:**
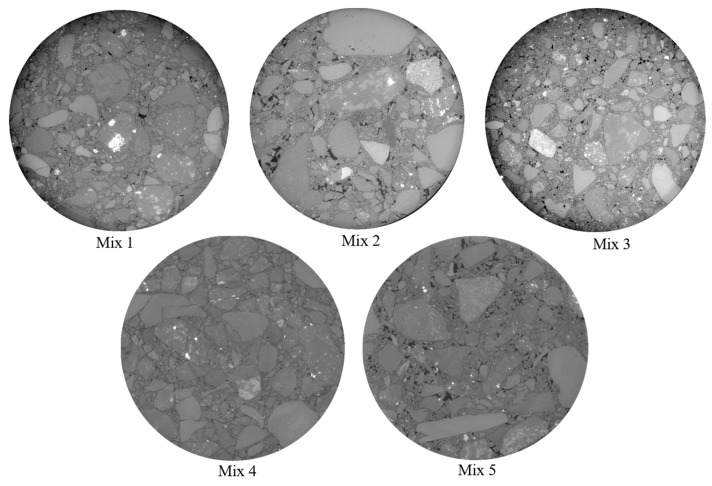
An example of 2D X-ray CT images for the tested AC mixtures at 0 F–T cycles.

The AC samples were scanned before F–T conditioning and after exposure to 30, 60, and 90 F–T cycles. The X-ray CT imaging was completed with a vertical interval of 0.016 mm and a horizontal resolution of 0.013 mm/pixel. Avizo software version 2021.2 was used to complete the reconstruction and register the 2D scans of samples before and after conditioning. ImageJ software version 2.9.0/1.54f (fiji) was used to analyze the registered 2D images. Figure 7 shows an example of the 2D X-ray CT images.

## 3. Data Analysis and Results

### 3.1. Change in Samples Volume

**Figure 8:**
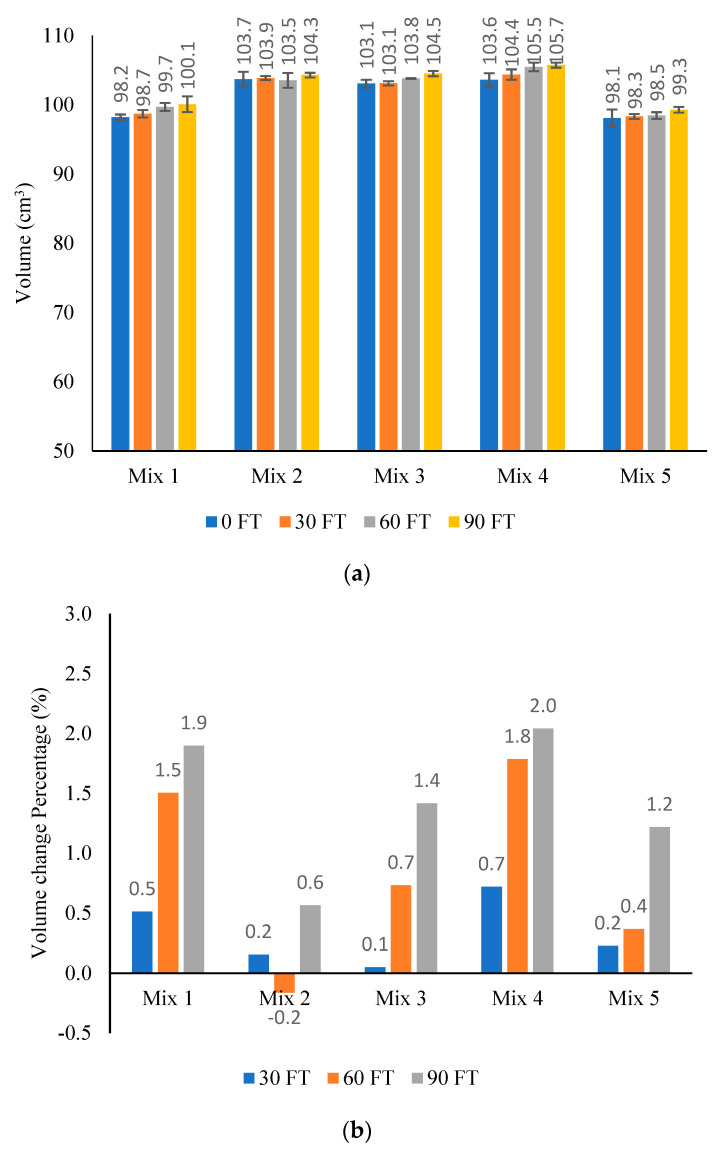
(**a**) Volume of AC samples before and after F–T cycling; (**b**) % change in samples volume.

Figure 8 shows the measured volume of AC samples at each F–T cycling stage and the percent of volume change due to 30, 60, and 90 F–T cycles. The results show that the volume of samples increased with the increase in the number of F–T cycles. After 90 F–T, Mix 1 and Mix 4 showed the highest volume change, 1.9% and 2% respectively, while Mix 2 showed the lowest volume change (0.6%). Mix 3 and Mix 5 showed a volume change of 1.4% and 1.2%, respectively, after 90 F–T cycles.

### 3.2. X-ray CT Scan Image Analysis

**Figure 9:**
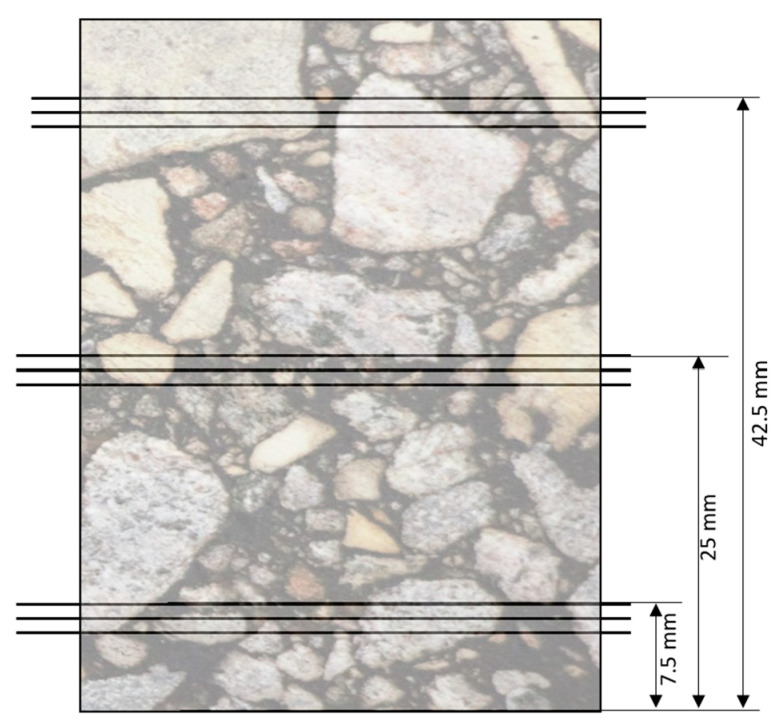
Locations of the selected slices from X-ray CT scans.

The samples were scanned before F–T cycling to get their initial internal structures. Samples were rescanned after 30, 60, and 90 F–T cycles to evaluate the changes in the internal structure due to F–T cycling. Nine 2D X-ray slices were taken from the bottom, middle, and top of each sample. The first three slices were taken at a height of 7.5 mm from the bottom of the sample, the second three slices were taken from the middle of the sample at a height of 25 mm, and the last three slices were taken from the top of the sample at a height of 42.5 mm (see Figure 9). These nine 2D X-ray slices were selected to represent the overall internal structure of the sample. The slices were analyzed using ImageJ software and Python version 3.7 to evaluate the properties of the internal voids in the samples. Table 3 shows the measured parameters from the image analysis to describe the changes in the internal air voids of samples.

**Figure 10:**
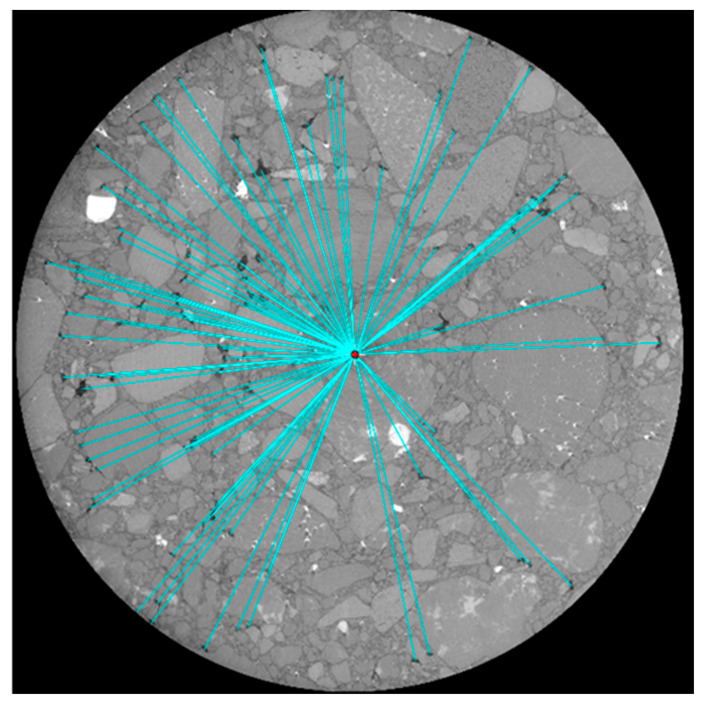
The distance between the centroids of air voids and the centroid of the sample.

Figure 10 shows an example of the distances between the centroid of air voids and the centroid of the sample in an X-ray slice. Measuring the mean of these distances (DVM) for different mixtures and after exposure to F–T cycling helps to understand the change in air void distribution, which can affect the F–T durability of AC.

#### 3.2.1. Image Analysis for Mix 1

Mix 1 is the conventional AC mix with the typical mix design used in Saskatchewan’s roads. Table 4 shows the measured parameters/properties of air voids for Mix 1. The image analysis results showed a formation of new air voids and an increase in the size of existing air voids after F–T cycling. The total number of air voids, the total area of air voids, and the air void percentage increased after 90 F–T cycles.

**Figure 11:**
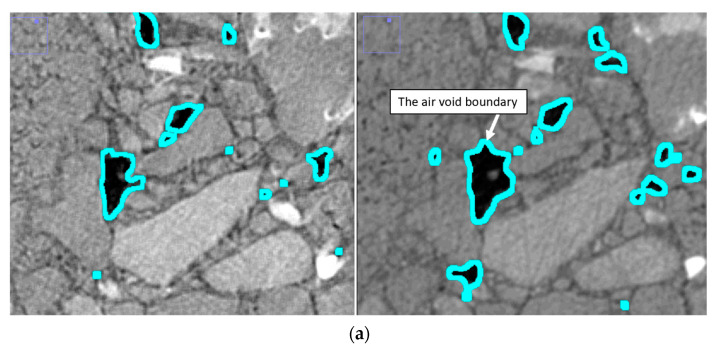

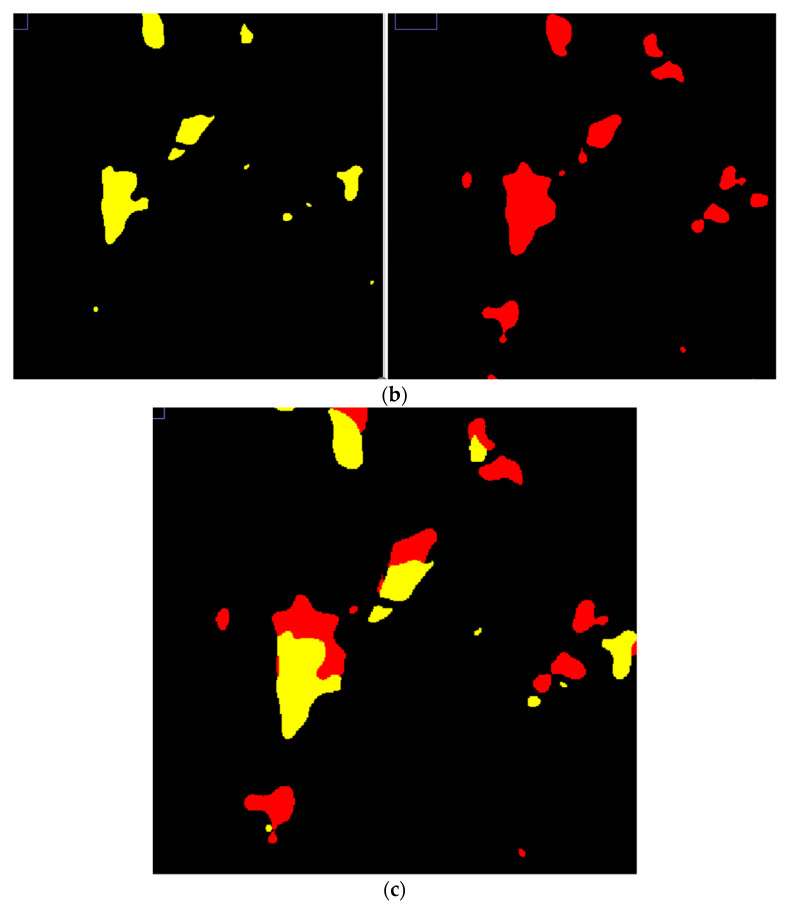
An example from Mix 1 showing the formation of new air voids and enlargement of the existing air voids after 90 F–T cycles: (**a**) X-ray CT scans for Mix 1 sample at 0 F–T cycles (left) and 90 F–T cycles (right); (**b**) ImageJ masks for Mix 1 sample at 0 F–T cycles (left) and 90 F–T cycles (right); (**c**) The original air voids at 0 F–T (yellow) with the new air voids (red) after 90 F–T.

**Figure 12:**
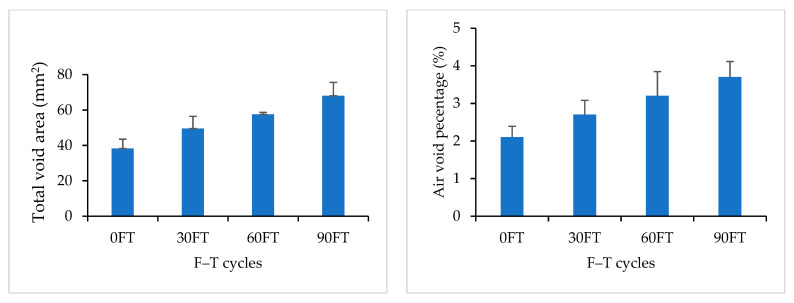

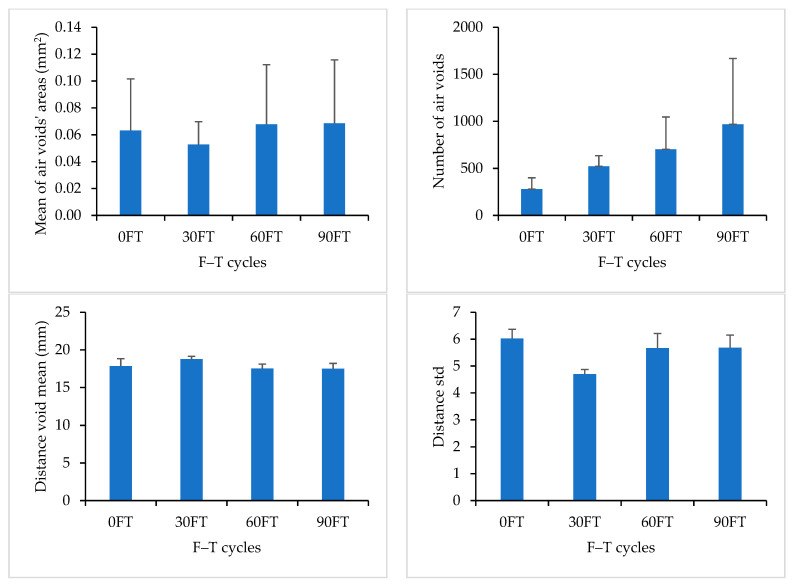
Image Analysis Results for Mix 1.

New, smaller air voids were formed at the bottom of the sample with the increase in F–T cycles. The existing air voids at the middle and top of the sample experienced enlargement and/or merged with adjacent air voids with the increase in F–T cycles, as explained in Figure 11. Figure 12 compares the measured properties of air voids for Mix 1 at 0, 30, 60, and 90 F–T cycles. The error bars represent the stranded deviation of the measured parameters, which represent their variability across the full height of the sample. Error bars are shown in positive values only for figure clarity. DVM values were comparable for all F–T cycling stages, which indicates that the air voids maintained a comparable distribution after 90 F–T cycles.

#### 3.2.2. Image Analysis for Mix 2

Mix 2 is a conventional mix with 20% recycled asphalt pavement (RAP) and polymer-modified asphalt binder. Table 5 shows the measured parameters/properties of air voids for Mix 2. The image analysis results for Mix 2 showed that the number of air voids was comparable after 90 F–T cycles. However, after 90 F–T cycles, the total area of air voids increased, which indicates an increase in the size of the initial air voids.

**Figure 13:**
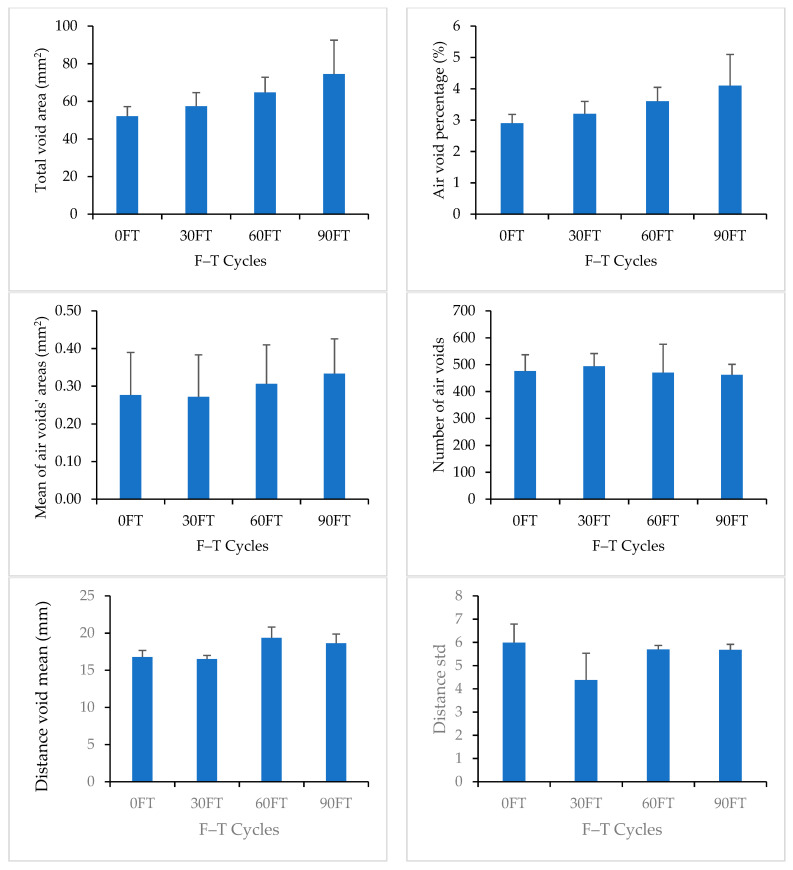
Image Analysis Results for Mix 2.

Figure 13 compares the measured properties of air voids for Mix 2 at 0, 30, 60, and 90 F–T cycles. DVM values increased by 11% after 90 F–T cycles. This increase can be attributed to the increase in air void size and shifting of their centroid locations.

#### 3.2.3. Image Analysis for Mix 3

Mix 3 contained a higher RAP content of 55%. Table 6 shows the measured properties of air voids for Mix 3. The image analysis results showed a formation of new air voids and an increase in the size of existing air voids due to F–T cycling. The total number of air voids, the total area of air voids, and the air void percentage increased after 90 F–T cycles.

**Figure 14:**
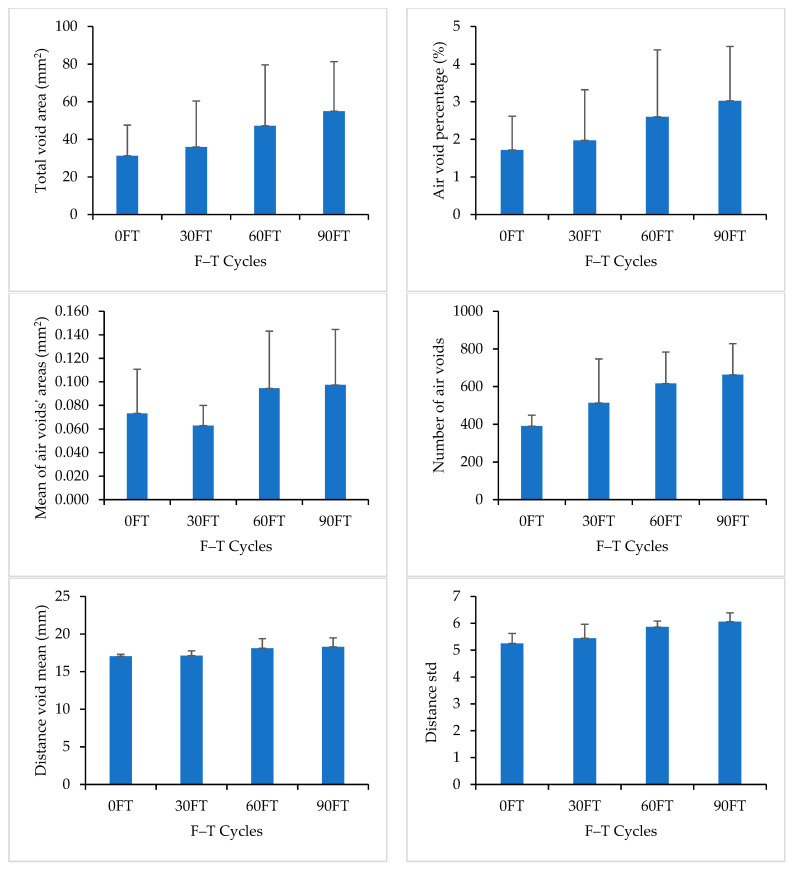
Image Analysis Results for Mix 3.

Figure 14 compares the measured properties of air voids for Mix 3 at 0, 30, 60, and 90 F–T cycles. DVM and DSTD values increased with the increase in the number of F–T cycles, which indicates an increase in air void scattering. This increase in air void scattering can be attributed to the formation of new air voids and the increase in existing air void size.

#### 3.2.4. Image Analysis for Mix 4

For Mix 4, the asphalt binder was modified with crumb rubbers with a content of 17.5% by weight. Table 7 shows the measured properties of air voids for Mix 4. The results showed a formation of new air voids and an increase in the size of existing air voids after F–T cycling. The total area of air voids, the air void percentage, and the mean size of air voids increased after 90 F–T cycles. Additionally, the number of air voids decreased after 90 F–T cycles, which can be attributed to the merging of adjacent air voids.

**Figure 15:**
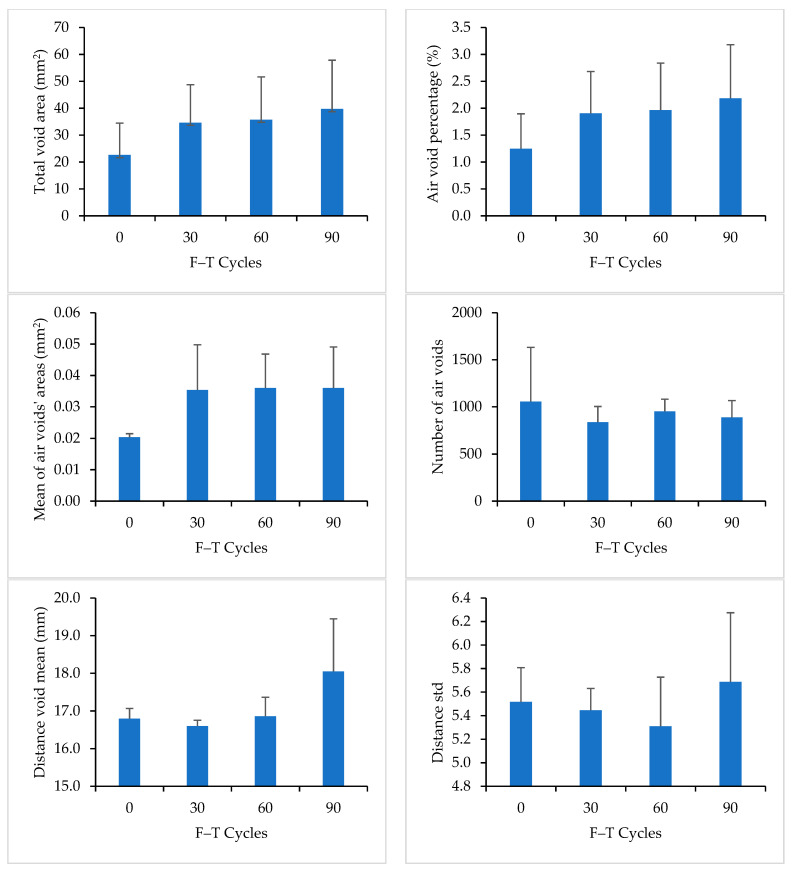
Image Analysis Results for Mix 4.

Figure 15 compares the measured properties of air voids for Mix 4 at 0, 30, 60, and 90 F–T cycles. DVM values increased with the increase in the number of F–T cycles. DSTD values were comparable for stages of F–T cycling, which indicates that the air voids maintained a comparable scattering after 90 F–T cycles.

#### 3.2.5. Image Analysis for Mix 5

Mix 5 contained a low RAP content of 8%. Table 8 shows the measured properties of air voids for Mix 5. The total number of air voids, the total area of air voids, and the air void percentage increased after 90 F–T cycles, which indicates the formation of new air voids due to F–T cycling.

**Figure 16:**
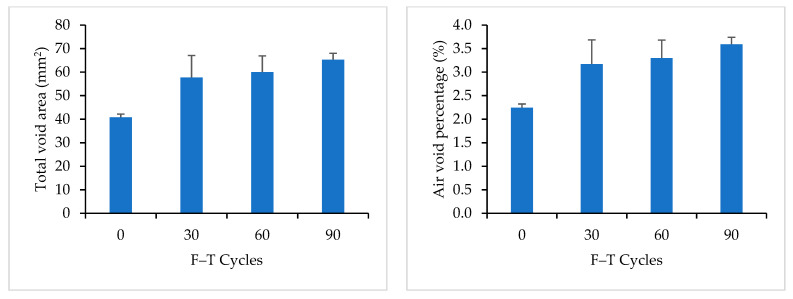

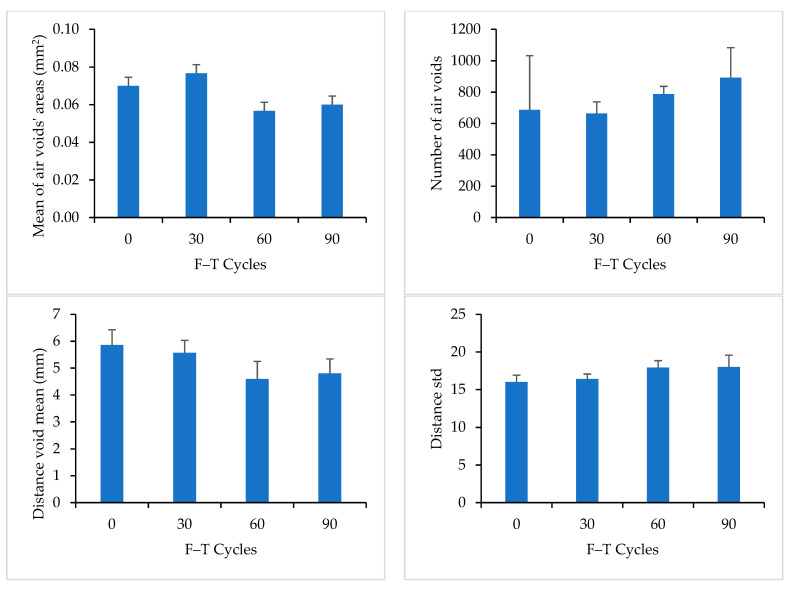
Image Analysis Results for Mix 5.

Figure 16 compares the measured properties of air voids for Mix 5 at 0, 30, 60, and 90 F–T cycles. DVM values increased with the increase in the number of F–T cycles, but the air voids were less scattered after 90 FT cycles.

## 4. Discussion

The composition of an AC mixture affects its F–T performance. There were differences in the composition of the five AC mixtures that were evaluated in this study. Mix 2 had a different type of asphalt binder, polymer-modified binder, while the remaining mixtures had the same asphalt binder grade. Mix 4 contained crumb rubbers, with a content of 17.5% by weight of binder. Mix 2, Mix 3, and Mix 5 contained RAP with a content of 20%, 55%, and 8%, respectively.

Although the aggregate gradations were within the specification limits for the five mixtures, Mix 4 had a coarser aggregate gradation than the other mixtures. Mix 2 and Mix 3 had finer aggregate gradations than the other mixtures, with more sand materials.

Table 9 summarizes the properties of the air voids’ properties for the five AC mixtures before exposure to F–T cycling. Mix 2 had the highest percentage of air voids (2.90%) with the largest mean size of air voids (0.28 mm^2^). The polymer-modified binder and the finer gradation of Mix 2 may have contributed to this distinct structure of air voids in the compacted samples.

Mix 4 had the lowest percentage of air voids (1.25%) with the smallest mean size of air voids (0.02 mm^2^). The crumb rubbers and the coarser aggregate gradation in Mix 4 may have contributed to this distinct structure of air voids in the compacted samples. The percentage of air voids and mean size of air voids were comparable for Mix 1 and Mix 5, which can be attributed to the comparable composition of these two mixtures (Mix 5 had 8% RAP only).

Table 10 shows the changes in the properties of air voids after 90 F–T cycles. Mix 1, Mix 3, and Mix 4 had the highest change in total air void area and percentage (increased by 75.3 to 78.1% of initial area), while Mix 2 had the lowest change in total air void area and percentage (increased by 42.9% of initial area). This pattern is comparable to the observed changes in sample volume, as shown in Figure 8.

For Mix 1, Mix 3, and Mix 5, the number of air voids increased after 90 F–T cycles. However, the number of air voids decreased in Mix 2 and Mix 4. Mix 1 had the highest increase in the number of air voids (increased by 247% of the initial number), while Mix 4 had the highest reduction in the number of air voids (reduced by 16% of the initial number).

Mix 4 had the highest increase in the average size of air voids (increased by 77.1% of the initial size), while Mix 5 had no change/reduction in the average size of air voids (reduced by 14% the of initial size). Mix 1, Mix 2, and Mix 3 had an increase of 8.4%, 20.7%, and 33.1%, respectively, of the initial average size of air voids.

DVM and DSTD explain the distribution and scattering of air voids. These parameters together with the change in the number and size of air voids can help to understand the mechanism of change in air void structure after F–T cycling (i.e., formation of new air voids, enlargement/merging of existing air voids, or combination of both), as discussed in the previous section. After 90 F–T cycles, Mix 5 and Mix 2 had the highest increase in the mean distances between the air voids’ centroids and the sample centroid (12.4% and 11.0% from the initial values). This pattern can be attributed to the formation of new air voids at the edges of the samples. For DSTD, Mix 3 had the highest increase in scattering (increased by 15% of the initial value), while Mix 5 had the highest decrease in scattering (decreased by 18% of the initial value).

For the test AC mixtures, the results showed varying mechanisms for changes in air void structure due to F–T cycling. The varying behavior of AC mixtures can be attributed to their varying compositions, which agrees with findings in the literature.

For mixtures with modified binders (Mix 2 and Mix 4), the increase in the total area of air voids after F–T cycling was dominated by the enlargement and merging of existing voids, rather than the formation of new air voids. For the conventional mixture (Mix 1) with no RAP, the increase in the total area of air voids after F–T cycling was dominated by the formation of new air voids with a limited increase in the size of existing voids.

For the 55% RAP mixture (Mix 3), the increase in the total area of air voids after F–T cycling was due to both the formation of new air voids and the enlargement/merging of existing air voids. There was no significant trend observed for the impact of increasing percentage of RAP on the structure of air voids after F–T cycling.

Mix 2, with a polymer-modified binder, had the largest size of air voids (MAV) at 0 F–T cycles. Having a larger size of air voids before F–T cycling contributed to the volume stability of this mixture, where Mix 2 had the lowest volume change after 90 F–T cycles.

## 5. Conclusions

Five AC mixtures were evaluated using X-ray CT scan imaging. Mix 1 is the conventional AC mixture used in Saskatchewan, Canada. Mix 2 contains a polymer-modified binder and 20% RAP. Mix 3 and Mix 5 contain 8% RAP and 55% RAP, respectively. Mix 4 contains a crumb-rubber-modified binder. The five AC mixtures were evaluated before conditioning and after exposure to 30, 60, and 90 F–T cycles. Samples were exposed to vacuum saturation before F–T cycling.

Based on the measurements of sample volume after 90 F–T cycles, Mix 1 and Mix 4 had the highest volume change (1.9% and 2.0%, respectively), while Mix 2 had the lowest volume change (0.6%). Mix 3 and Mix 5 showed a volume change of 1.4% and 1.2%, respectively.

The results of image analysis indicate that Mix 2 was less susceptible to F–T cycling than other mixtures (in terms of changes in the total area of air voids, air void percentage, and formation of new air voids). These results agree with the observations from the volume measurements of the AC samples. The volume and air void stability of Mix 2 after F–T cycling can be attributed to the use of a polymer-modified binder in the mixture.

The AC mixtures showed different mechanisms for changes in air void structure after F–T cycling. For Mix 2 and Mix 4, the change in the total area of air voids after F–T cycling was dominated by the enlargement and/or merging of initial air voids. This behavior can be attributed to the use of polymer-modified and crumb-rubber-modified binders in these two mixtures. For Mix 1 and Mix 5, the change in the total area of air voids after F–T cycling was dominated by the formation of new air voids. For Mix 3, the change in the total area of air voids after F–T cycling was due to both the formation of new air voids and the enlargement and/or merging of existing air voids.

## Figures and Tables

**Table 1:** Properties of AC mixtures.

Mix ID	Mix 1	Mix 2	Mix 3	Mix 4	Mix 5
Description	Conventional	20% RAP	55% RAP	17.5% crumb rubber by weight of binder	8% RAP
Binder type	200-300A (PG52-34)	PG 58-37P	150-200A (PG52-34)	PG52-34	PG52-34
Nominal maximum aggregate size (NMAS)	12.5 mm	12.5 mm	12.5 mm	12.5 mm	12.5 mm
Asphalt binder percentage %	5.4	5.5	5.4	7.3	5.4
Air void percentage %	3.8	3.8	4	3.4	4
Compaction Temp °C	131	148	125	165	134
No. of Gyrations	115	93	79	114	103

**Table 2:** Volumetric properties of the lab-compacted samples.

Volumetric Properties	Mix 1		Mix 2		Mix 3		Mix 4		Mix 5	
	Mix Design	Lab Value	Mix Design	Lab Value	Mix Design	Lab Value	Mix Design	Lab Value	Mix Design	Lab Value
G_mb_	2.384	2.397	2.406	2.439	2.400	2.429	2.354	2.334	2.393	2.427
G_mm_	2.477	2.499	2.502	2.544	2.502	2.504	2.437	2.405	2.493	2.507
VA (%)	3.8	4.1	3.8	4.1	4.0	3.09	3.4	2.9	4.0	3.2
VMA (%)	14.3	14.0	14.4	13.4	15.0	14.4	17.3	18.4	14.6	13.7
VFA (%)	73.6	70.9	73.4	69.2	73.1	79.2	80.3	84.1	72.8	76.9

**Table 3:** Image Analysis Measurements.

Parameter	Description and Units
Condition	Freeze–thaw stage (0 FT, 30 FT, 60 FT, and 90 FT).
Total voids area (TVA)	The mean of air void total area in the analyzed X-ray slices (mm^2^).
Air void percentage (AV)	The mean of air void percentage in the analyzed X-ray slices (%).
Mean area of air voids (MAV)	The mean area of air voids in the analyzed X-ray slices (mm^2^).
Number of air voids (NAV)	The mean of air voids in the analyzed X-ray slices.
Distance void mean (DVM)	The mean of the distances between the air void’s centroid and the sample’s centroid in the analyzed X-ray slices (mm), the air void’s distribution, see Figure 10.
Distance std (DSTD)	The mean of the standard deviation of the distances between the air void’s centroid and the sample’s centroid in the analyzed X-ray slices.

**Table 4:** Air voids properties for Mix 1.

No. of F–T Cycles	0		30		60		90	
	Avg	Std	Avg	Std	Avg	Std	Avg	Std
TVA (mm^2^)	38.224	5.288	49.498	6.921	57.559	1.173	68.081	7.550
AV (%)	2.100	0.003	2.700	0.004	3.200	0.001	3.700	0.004
MAV (mm^2^)	0.063	0.038	0.053	0.017	0.068	0.044	0.069	0.047
NAV	279	120	522	114	702	344	969	699
DVM (mm)	17.834	0.992	18.777	0.376	17.518	0.595	17.495	0.713
DSTD	6.024	0.343	4.695	0.173	5.669	0.544	5.678	0.471

**Table 5:** Air void size properties for Mix 2.

No. of F–T Cycles	0		30		60		90	
	Avg	Std	Avg	Std	Avg	Std	Avg	Std
TVA (mm^2^)	52.110	5.098	57.400	7.225	64.706	8.120	74.453	18.094
AV (%)	2.900	0.281	3.200	0.398	3.600	0.448	4.100	0.998
MAV (mm^2^)	0.276	0.113	0.272	0.112	0.306	0.103	0.333	0.092
NAV	476	61	494	47	470	106	462	39
DVM (mm)	16.778	0.882	16.515	0.478	19.353	1.457	18.630	1.244
DSTD	5.986	0.805	4.377	1.158	5.698	0.168	5.678	0.237

**Table 6:** Air void properties for Mix 3.

No. of F–T Cycles	0		30		60		90	
	Avg	Std	Avg	Std	Avg	Std	Avg	Std
TVA (mm^2^)	31.238	16.321	35.866	24.490	47.216	32.398	54.959	26.366
AV (%)	1.7	0.9	2.0	1.3	2.6	1.8	3.0	1.4
MAV (mm^2^)	0.073	0.038	0.063	0.017	0.094	0.049	0.097	0.047
NAV	391	57	513	234	617	167	663	164
DVM (mm)	17.050	0.256	17.112	0.648	18.097	1.294	18.299	1.194
DSTD	5.249	0.371	5.439	0.524	5.862	0.220	6.059	0.331

**Table 7:** Air void properties for Mix 4.

No. of F–T Cycles	0		30		60		90	
	Avg	Std	Avg	Std	Avg	Std	Avg	Std
TVA (mm^2^)	22.636	11.838	34.602	14.157	35.729	15.889	39.685	18.171
AV (%)	1.245	0.651	1.903	0.779	1.965	0.874	2.182	0.999
MAV (mm^2^)	0.020	0.001	0.035	0.014	0.036	0.011	0.036	0.013
NAV	1055	576	836	167	951	129	886	179
DVM (mm)	16.796	0.273	16.598	0.154	16.861	0.503	18.046	1.402
DSTD	5.516	0.292	5.446	0.185	5.310	0.417	5.686	0.588

**Table 8:** Air void properties for Mix 5.

No. of F–T Cycles	0		30		60		90	
	Avg	Std	Avg	Std	Avg	Std	Avg	Std
TVA (mm^2^)	40.79	1.419	57.68	9.364	60.00	6.918	65.27	2.729
AV (%)	2.24	0.080	3.17	0.514	3.30	0.384	3.59	0.149
MAV (mm^2^)	0.07	0.010	0.08	0.038	0.06	0.021	0.06	0.036
NAV	687	346	664	75	787	50	892	191
DVM (mm)	16.01	0.917	16.41	0.656	17.92	0.921	17.99	1.583
DSTD	5.86	0.566	5.57	0.467	4.59	0.657	4.80	0.536

**Table 9:** Air void properties before conditioning (at 0 F–T cycles).

	Mix 1	Mix 2	Mix 3	Mix 4	Mix 5
TVA (mm^2^)	38.22	52.11	31.24	22.64	40.79
AV (%)	2.10	2.90	1.70	1.25	2.24
MAV (mm^2^)	0.06	0.28	0.07	0.02	0.07
NAV	279	476	391	1055	687
DVM (mm)	17.834	16.778	17.050	16.796	16.01
DSTD	6.024	5.986	5.249	5.516	5.86

**Table 10:** The changes in air void properties after 90 F–T cycles (absolute values and percentage of initial values).

	Mix 1		Mix 2		Mix 3		Mix 4		Mix 5	
	Avg. Diff.	%	Avg. Diff.	%	Avg. Diff.	%	Avg. Diff.	%	Avg. Diff.	%
TVA (mm^2^)	29.9	78.1	22.3	42.9	23.7	75.9	17.1	75.3	24.5	60.0
AV (%)	1.60	76.2	1.2	41.4	1.3	75.9	0.9	75.3	1.4	60.0
MAV (mm^2^)	0.01	8.4	0.06	20.7	0.02	33.1	0.02	77.1	−0.01	−14.3
NAV	690	247	−14	−3	273	70	−169	−16	205	30
DVM (mm)	−0.34	−1.0	1.85	11.0	1.25	7.3	1.25	7.4	1.99	12.4
DSTD	−0.35	−5.7	−0.31	−5.2	0.81	15.4	0.17	3.1	−1.06	−18.0

Avg. diff. is the difference between the absolute values at 0 F–T and 90 F–T. % is the change percentage of initial values at 0 F–T cycles.

## Data Availability

Details of the data are available from the corresponding author.
